# The Formation of Stable Lung Tumor Spheroids during Random Positioning Involves Increased Estrogen Sensitivity

**DOI:** 10.3390/biom14101292

**Published:** 2024-10-12

**Authors:** Balkis Barkia, Viviann Sandt, Daniela Melnik, José Luis Cortés-Sánchez, Shannon Marchal, Bjorn Baselet, Sarah Baatout, Jayashree Sahana, Daniela Grimm, Markus Wehland, Herbert Schulz, Manfred Infanger, Armin Kraus, Marcus Krüger

**Affiliations:** 1 Department of Microgravity and Translational Regenerative Medicine, Otto von Guericke University, 39106 Magdeburg, Germany; balkissbarkia@gmail.com (B.B.); viviann.sandt@med.ovgu.de (V.S.); daniela.melnik@med.ovgu.de (D.M.); jose.cortes@ovgu.de (J.L.C.-S.); shannon.marchal@med.ovgu.de (S.M.); markus.wehland@med.ovgu.de (M.W.); herbert.schulz@med.ovgu.de (H.S.); 2 Radiobiology Unit, Belgian Nuclear Research Centre SCK-CEN, 2400 Mol, Belgium; bjorn.baselet@sckcen.be (B.B.); sarah.baatout@sckcen.be (S.B.); 3 Department of Biotechnology, Faculty of Bioscience Engineering, Ghent University, 9000 Ghent, Belgium; 4 Department of Biomedicine, Faculty of Health, Aarhus University, 8000 Aarhus, Denmark; jaysaha@biomed.au.dk (J.S.); dgg@biomed.au.dk (D.G.); 5 Research Group “Magdeburger Arbeitsgemeinschaft für Forschung unter Raumfahrt- und Schwerelosigkeitsbedingungen” (MARS), Otto von Guericke University, 39106 Magdeburg, Germany; manfred.infanger@med.ovgu.de (M.I.); armin.kraus@med.ovgu.de (A.K.); 6 Clinic for Plastic, Aesthetic and Hand Surgery, University Hospital Magdeburg, 39120 Magdeburg, Germany

**Keywords:** lung cancer, tumor spheroids, simulated microgravity, cell signaling, phenol red

## Abstract

The formation of tumor spheroids on the random positioning machine (RPM) is a complex and important process, as it enables the study of metastasis ex vivo. However, this process is not yet understood in detail. In this study, we compared the RPM-induced spheroid formation of two cell types of lung carcinoma (NCI-H1703 squamous cell carcinoma cells and Calu-3 adenocarcinoma cells). While NCI-H1703 cells were mainly present as spheroids after 3 days of random positioning, Calu-3 cells remained predominantly as a cell layer. We found that two-dimensional-growing Calu-3 cells have less mucin-1, further downregulate their expression on the RPM and therefore exhibit a higher adhesiveness. In addition, we observed that Calu-3 cells can form spheroids, but they are unstable due to an imbalanced ratio of adhesion proteins (β_1_-integrin, E-cadherin) and anti-adhesion proteins (mucin-1) and are likely to disintegrate in the shear environment of the RPM. RPM-exposed Calu-3 cells showed a strongly upregulated expression of the estrogen receptor alpha gene *ESR1*. In the presence of 17β-estradiol or phenol red, more stable Calu-3 spheroids were formed, which was presumably related to an increased amount of E-cadherin in the cell aggregates. Thus, RPM-induced tumor spheroid formation depends not solely on cell-type-specific properties but also on the complex interplay between the mechanical influences of the RPM and, to some extent, the chemical composition of the medium used during the experiments.

## 1. Introduction

The random positioning machine (RPM) is a tool originally from gravitational biology and space medicine that can be used to research new ways of fighting cancer [1,2]. The RPM has already been used to study various types of cancer, including thyroid, pancreatic, breast and prostate cancer [3,4,5,6,7,8,9,10,11]. One effect that has been observed in all cancer cells examined on the RPM so far is the detachment of cells from the cell layer and the subsequent formation of three-dimensional tumor spheroids. This process, which shows molecular biological similarity to metastasis in vivo, has recently led to the designation of the RPM culture of cancer cells as an in vitro metastasis model [12,13,14]. However, the morphology and quality of tumor spheroid formation depends on the cell type used and its response to the RPM cell culture environment. MCF-7 breast cancer cells, for example, form glandular spheroids [11], while FTC-133 thyroid tumor spheroids show compact aggregation [10]. The spheroids from PC-3 prostate carcinoma cells are somewhat unstable and loose during the first few days on the RPM [3]. The amount of expressed membrane glycoproteins such as the anti-adhesive mucin-1 also has an influence on the number of spheroids formed by random positioning from ML-1 thyroid carcinoma cells and LnCAP prostate carcinoma cells [14].

Lung carcinoma cells have rarely been examined on the RPM. However, lung cancer is one of the most common and deadliest type of cancer worldwide. According to the latest GLOBOCAN data, lung cancer now ranks first in both incidence (12.4% of all sites) and mortality (18.7% of all sites), ahead of breast, colorectal and prostate cancer [15]. New therapeutic strategies and in vitro approaches for more detailed research are therefore urgently needed, as the prognosis is particularly poor for non-small cell lung cancer (NSCLC) patients, who account for 85% of lung cancer cases [16]. Lung cancer usually only becomes noticeable when it has spread to a high degree in the lungs or metastasized to other parts of the body. When lung carcinomas are discovered, they are usually at metastatic stage IIIB or IV [16,17]. During metastasis, there are two different forms of lung tumor cell migration: the movement of single cells or small cell clusters, as seen in small cell carcinomas (SCLCs) and undifferentiated NSCLCs, and the movement of large clusters of organized cells, as typically seen in adenocarcinomas or some cases of squamous cell carcinomas (both NSCLCs) [17]. The organized cell clusters, which were similarly observed as tumor spheroids in RPM experiments for other types of cancer, suggest a more detailed investigation in the “in vitro metastasis model”. Following the pilot study by Ahn et al. [18], which showed that the migration rate of two-dimensional-growing A549 cells under random positioning was faster than that of NCI-H1703, we are the second team to compare cells from a squamous cell carcinoma with cells from an adenocarcinoma on the RPM; this time focusing on tumor spheroids.

## 2. Materials and Methods

### 2.1. Cell Lines and Cell Culture

The NCI-H1703 lung squamous cell carcinoma cell line (passages 20–28) was purchased from ATCC (Manassas, VA, USA). The Calu-3 lung adenocarcinoma cell line (passages 16–25) was a kind gift from Prof. Heike Walles, University of Magdeburg. Both cell lines were cultured in phenol-red-free DMEM/F12 medium + 2.5 mM L-glutamine + 29 mM sodium bicarbonate (Life Technologies, Carlsbad, CA, USA) supplemented with 10% fetal calf serum (FCS; Sigma-Aldrich, St. Louis, MO, USA) and 1% penicillin/streptomycin (Life Technologies) at 37 °C, 5% CO_2_ and a relative humidity of 95%. An identical DMEM/F12 medium with phenol red (Life Technologies) was used for comparative experiments to study the effects of phenol red. Cells were routinely checked microscopically and passaged at a maximum confluence of 90%. For the experiments, a cell density of 0.5 × 10^6^ cells was seeded into uncoated T25 cell culture flasks (Sarstedt, Nümbrecht, Germany) and given 24 h for the cells to settle and adhere. For immunofluorescence staining, uncoated glass coverslips (Carl Roth, Karlsruhe, Germany) with sterilized Vaseline (Edeka, Hamburg, Germany) were placed in a T25 flask, which were then UV illuminated for at least one hour and allowed to dry overnight before seeding the cells.

### 2.2. Random Positioning

For most experiments, a desktop RPM 2.0 (Yuri, Meckenbeuren, Germany) was used in a HERAcell CO_2_ incubator (Thermo Scientific, Waltham, MA, USA) at 37 °C, 5% CO_2_ without humidification. An incubator RPM (iRPM 2.0; developed by Prof. Jörg Sekler, University of Applied Sciences and Arts Northwestern Switzerland [19]) was used for comparison experiments, which offered a larger sample load for the analyses (e.g., gene expression analysis). Both devices led to the same phenotypic observations and are therefore comparable for this study. The medium was not specially pretreated. For random positioning, the RPM was operated in real random mode (two frames rotation) at an average speed of 60°/s (range: 50–70°/s). Before starting the rotation, the cell culture flasks were filled completely with medium, avoiding bubbles (any remaining bubbles were carefully removed using a pipette tip before closing the culture flask). Static controls were placed next to the desktop RPM or in the same environmental conditions (iRPM experiments).

### 2.3. Static Spheroid Formation

Cells were seeded in 96-well BIOFLOAT™ U-bottom plates (faCellitate, Mannheim, Germany) at a density of 8000 cells per well cultured in phenol red and phenol-red-free medium at 37 °C and 5% CO_2_. Images were captured every 24 h over seven days using an Olympus CKX53 inverted microscope (Olympus, Tokyo, Japan).

### 2.4. Estrogen Treatment (Calu-3)

17β-estradiol (Sigma-Aldrich) was dissolved in 100% ethanol (Chemsolute; Th. Geyer, Renningen, Germany). To tests the effects of 17β-estradiol, we supplemented phenol-red-free DMEM/12 medium with 10 nM 17β-estradiol from the beginning of cell culture.

### 2.5. mRNA Isolation and Quantitative Real-Time PCR

The adherent cells were carefully washed once with DPBS (Life Technologies), fixed with RNAlater^TM^ solution (Invitrogen, Life Technologies) and harvested with a cell scraper. The spheroids were centrifuged at 125× *g* for 5 min; the supernatant was discarded and then fixed with RNAlater^TM^. Further processing of the samples was performed in the same way as described in [14]. The primer sequences used for the quantitative real-time PCR are listed in the Supplementary Materials (Table S1). The PCR reaction was performed on a QuantStudio™ 3 device (Thermo Fisher Scientific). Samples were measured in triplicate and evaluated by using the comparative threshold cycle (ΔΔC_T_) method with 18S rRNA as the housekeeper reference. QuantStudio™ Design & Analysis software (v1.5, Thermo Fisher Scientific) was used, which allows an internal check of the curve and result quality to detect misleading results that should be excluded. The individual data points are displayed in the graphs. Outliers in biological replicates were identified with the Grubbs’/ESD test using the GraphPad outlier calculator (Prism v9, GraphPad Software, Boston, MA, USA).

### 2.6. Luminex^®^ Multiplex Assay

Multiplex bead arrays for TNF, IL-6, IL-8 and osteopontin protein levels in the cell culture supernatant were analyzed using a multiplex magnetic bead array (Human LXSAHM-06; R&D systems, Minneapolis, MN, USA). Assays were performed according to the manufacturer’s instructions. Samples were run on a MAGPIX instrument (Luminex, Austin, TX, USA) and analyzed with Belysa Immunoassay Curve Fitting Software (v1.2, Sigma-Aldrich, St. Louis, MO, USA).

### 2.7. Phase Contrast Microscopy

Cells were monitored and imaged using an Olympus CKX53 inverted microscope (Olympus, Tokyo, Japan) in phase contrast mode with a magnification of 10× (NA: 0.25) or 20× (NA: 0.4). A Keyence BZ-X810 microscope (Keyence, Neu-Isenburg, Germany) in phase contrast mode at a magnification of 10× (NA: 0.45) was used to inspect and image the cells during the spheroid-formation experiment in 96-well plates. The image analysis was carried out with Fiji software (v1.54f, ImageJ, imagej.net).

### 2.8. Immunofluorescence Microscopy and Analysis

For fixation, the two-dimensional-growing cells were washed once with 0.1 M phosphate buffer (PB; Na_2_HPO_4_/NaH_2_PO_4_; Carl Roth, Karlsruhe, Germany) and fixed with 4% paraformaldehyde (PFA; Carl Roth). Spheroids were collected in centrifugation tubes (Sarstedt), centrifuged at 125× *g* for 5 min at 4 °C and fixed with 4% PFA. All samples were stored at 4 °C until the staining procedure (analyzed within 3 days). To remove the 4% PFA, the cells were washed three times with 0.1 PB. The cell membranes were permeabilized with 0.2% Triton X-100 (Carl Roth) in 0.1 M PB and washed three times with 0.1 M PB. To block non-specific binding sites, 3% bovine serum albumin (BSA, Carl Roth) in 0.1 M PB was used for 1 h at room temperature. Cells were then labeled with primary antibodies (Table S2) diluted in 0.1 M PB with 1% BSA overnight at 4 °C. The next day, the cells were washed three times with 0.1 M PB, incubated with the secondary antibodies (Table S2) for 1 h at room temperature and washed again three times with 0.1 M PB. For F-actin staining, Alexa Fluor™ 568 Phalloidin (Invitrogen #A12380, Life Technologies) was added at a dilution of 1:400 for 1 h. For nuclear DNA staining, the samples were incubated for 5–10 min with 100 ng/mL DAPI (4′,6-diamidino-2-phenylindole; Thermo Fisher Scientific) in 0.1 M PB, finally washed three times with 0.1 M PB and mounted with Fluoromount^TM^ (Sigma-Aldrich). The spheroids were treated accordingly. For spheroid staining, however, the cell aggregates were incubated with both the primary and secondary antibodies for 24 h. For nuclear DNA staining, the samples were incubated for 2 h at 4 °C with 100 ng/mL DAPI in 0.1 M PB. Washing steps for spheroids were accompanied by soft centrifugation at 200 rpm for 1 min at 4 °C.

After staining, the slides were examined using a ZEISS LSM 800 confocal laser scanning microscope (Carl Zeiss, Jena, Germany). To ensure comparability of the images, all the images were acquired with the same settings using the ZEISS Airyscan detector and ZEN 3.4 software (Carl Zeiss). The Airyscan processing settings were optimized for each antibody–wavelength combination. To check for non-specific binding of the secondary antibody and thus a false negative signal, the secondary antibodies were applied to separate samples of the same condition without the primary antibody.

### 2.9. Statistical Analysis

Statistical evaluation was performed using SPSS Statistics (v29, IBM, Armonk, NY, USA). The non-parametric Mann–Whitney U test was used to compare samples (biological replicates) from different culture conditions. Outliers were omitted before analysis. All data are presented as the mean ± standard deviation (SD). In most plots, the individual data points are shown additionally. The sample size for each experiment is provided in the figure legends.

### 2.10. Interaction Networks of Chemicals and Proteins (STITCH Analysis)

The interactions between phenol red and proteins were determined using the STITCH (‘search tool for interactions of chemicals’) v5.0 database, available at http://stitch.embl.de/ (accessed on 17 July 2024) [20]. The search was performed for *Homo sapiens* with a high confidence interaction score.

## 3. Results

### 3.1. Different Lung Cancer Types Show Different Spheroid-Formation Ability When Exposed to the Random Positioning Machine

**Figure 1:**
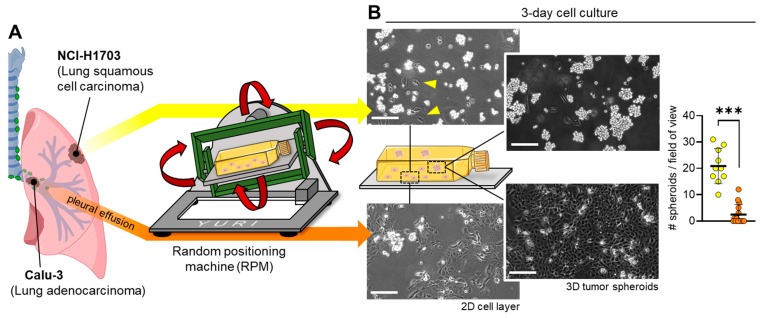
On the RPM, two-dimensional cultures of different lung carcinoma cell types resulted in different subpopulations. (**A**) While NCI-H1703 squamous cell carcinoma cells (yellow line, top) mainly formed 3D aggregates after 3 days of rotation (some of the few adherent cells are indicated by yellow arrows), Calu-3 adenocarcinoma cells (orange line, bottom) remained almost completely as a two-dimensional cell layer. (**B**) Microscopic images of the cell culture after 3 days of dynamic culture on the RPM. The bar chart shows the number of spheroids per microscopic image. Scale bar: 200 µm. Non-parametric Mann–Whitney U test *** *p* ≤ 0.001. Parts of the figure were drawn using pictures from Biorender.com and Servier Medical Art.

To compare the spheroid formation of carcinoma cells from different lung origin tissues, we cultured model cell lines of squamous cell carcinoma (NCI-H1703) and adenocarcinoma (Calu-3) for 3 days on the RPM (Figure 1A). At the beginning of the experiment, the cells were mounted as subconfluent 2D cultures. At the end of the experiment, the growth forms of the two dynamic cell cultures were very different: while the NCI-H1703 cells were mainly present as 3D spheroids, the Calu-3 cultures remained almost completely two-dimensional (Figure 1B).

### 3.2. Spheroid Stability Due to the Presence of Adhesion and Anti-Adhesion Proteins

**Figure 2:**
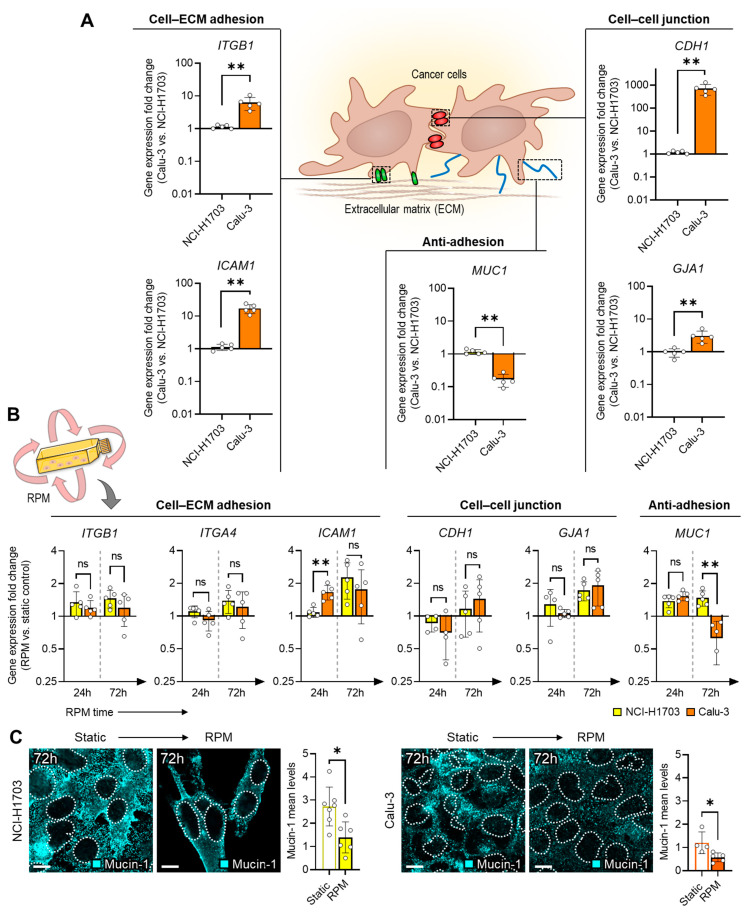
Gene expression differences of cell adhesion molecules in two-dimensional cell cultures of NCI-H1703 (orange) and Calu-3 cells (yellow). (**A**) Relative expression differences in Calu-3 vs. NCI-H1703 cells in a static cell culture after 24 h (*n* = 5). (**B**) Expression changes in a dynamic 2D RPM cell culture after 24 h and 72 h (*n* = 4–5). (**C**) Immunofluorescence of mucin-1 in a 3-day culture (*n* = 5 for each condition; one representative picture is shown). Outlines of the nuclei as indicated using DAPI staining depicted as dashed lines. The averaged mean values of mucin-1 fluorescence per cell for each microscopic image are shown in the bar graphs. Scale bar: 10 µm. Non-parametric Mann–Whitney U test * *p* ≤ 0.05, ** *p* ≤ 0.01, ^ns^ non-significant. Parts of the figure were drawn using pictures from Biorender.com and Servier Medical Art.

To better understand the different spheroid-formation behaviors, the gene expression of different cell–cell, cell–extracellular matrix (ECM) and anti-adhesion molecules was investigated. We have already recognized in a previous RPM study that the balance between adhesion and anti-adhesion proteins can influence RPM-induced spheroid formation [14]. At the transcription level, these factors were already present in different amounts in the two cell lines studied. In static cell culture, Calu-3 cells showed a higher basal expression in adhesion proteins (*ITGB1*, *ICAM1*, *CDH1*, *GJA1*), and a lower expression of the anti-adhesion gene *MUC1* compared to NCI-H1703 cells (Figure 2A).

The one-day and three-day cultivation on the RPM resulted in only minor differences in the expression of the respective genes between the two cell lines (Figure 2B). There was a significant reduction in *MUC1* expression in Calu-3 cells after 72 h on the RPM (Figure 2B), which may indicate that the cell layer of Calu-3 may be more adhesive on the RPM. Immunofluorescence analysis confirmed the slightly lower mucin-1 level in Calu-3 cells compared to NCI-H1703 cells and a reduction in mucin-1 in Calu-3 cells after 72 h on the RPM at the protein level (Figure 2C).

**Figure 3:**
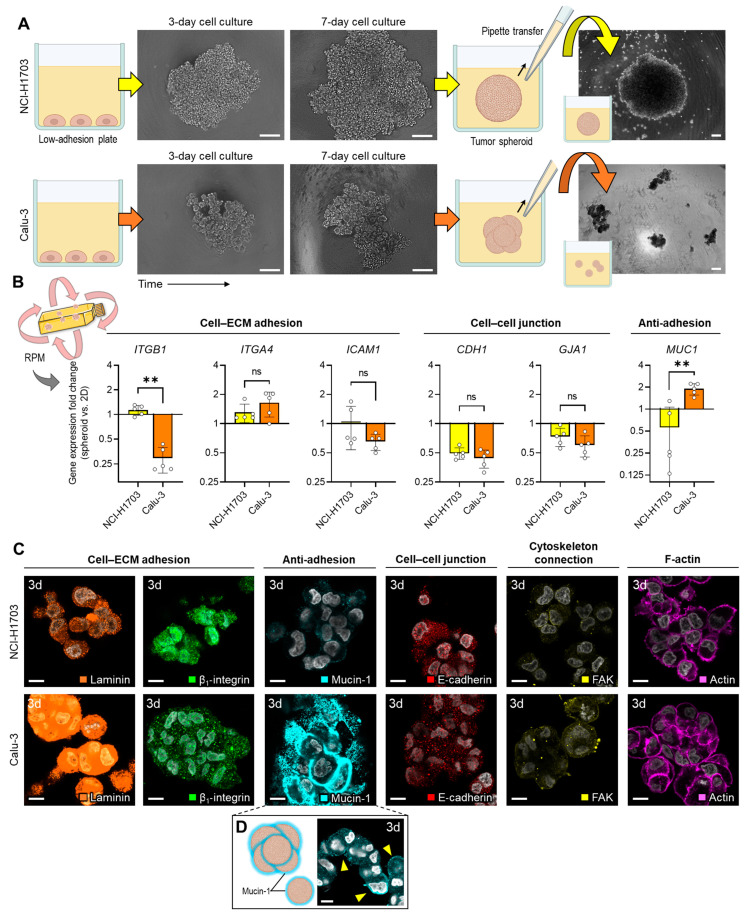
(**A**) Spheroid formation of NCI-H1703 and Calu-3 cells in low-adhesion plates within 7 days. The two cell lines show a different spheroid morphology. Mechanical influences (such as pipetting) led to the disintegration of 7-day Calu-3 tumor spheroids. Scale bar: 200 µm. (**B**) Gene expression changes in spheroid cells compared to 2D culture on RPM after 3 days (*n* = 5). (**C**) Immunofluorescence of laminin, β_1_-integrin, mucin-1, E-cadherin, focal adhesion kinase (FAK) and F-actin combination in a 3-day culture (*n* = 5 for each condition; one representative picture is shown). Nuclei are stained with DAPI (gray). Scale bar: 10 µm. (**D**) Typical mucin-1 distribution in a Calu-3 spheroid structure around the subaggregates (yellow arrows). Scale bar: 10 µm. Non-parametric Mann–Whitney U test ** *p* ≤ 0.01, ^ns^ non-significant. Parts of the figure were drawn using pictures from Biorender.com and Servier Medical Art.

Since we initially found no explanation for the different amounts of spheroids in the two cell types in the expression data, we next investigated the ability of the cells to form spheroids under static conditions by using a low-adhesion plate. Here, we observed that both cells are quite capable of forming 3D aggregates. While NCI-H1703 cells formed homogeneous spheroids in the plate, Calu-3 spheroids consisted of several small, “cauliflower-like” substructures (Figure 3A). When attempting to transfer the spheroids with a pipette, the instability of the Calu-3 spheroids quickly became apparent as they disintegrated into their substructures (Figure 3A). We assume that this also happened on the RPM, where higher shear forces occur inside the culture flasks [21,22,23].

Gene expression analysis of the spheroids showed that aggregated Calu-3 cells downregulate the adhesion-related genes *ITGB1* and *ICAM1*, while upregulating the anti-adhesion-related *MUC1* (Figure 3B). Consistent with the gene expression data, immunofluorescence staining of 3-day-old tumor spheroids from the RPM culture confirmed that Calu-3 spheroids are mainly characterized by less β_1_-integrin and E-cadherin and much more mucin-1 and laminin compared to NCI-H1703 spheroids at this stage (Figure 3C). “Cauliflower-like” Calu-3 spheroid aggregates showed a mucin-1 distribution around the subaggregates (Figure 3D), which easily fell apart under mechanical impact.

### 3.3. RPM-Induced Secretion of Multifunctional Signaling Molecules

**Figure 4:**
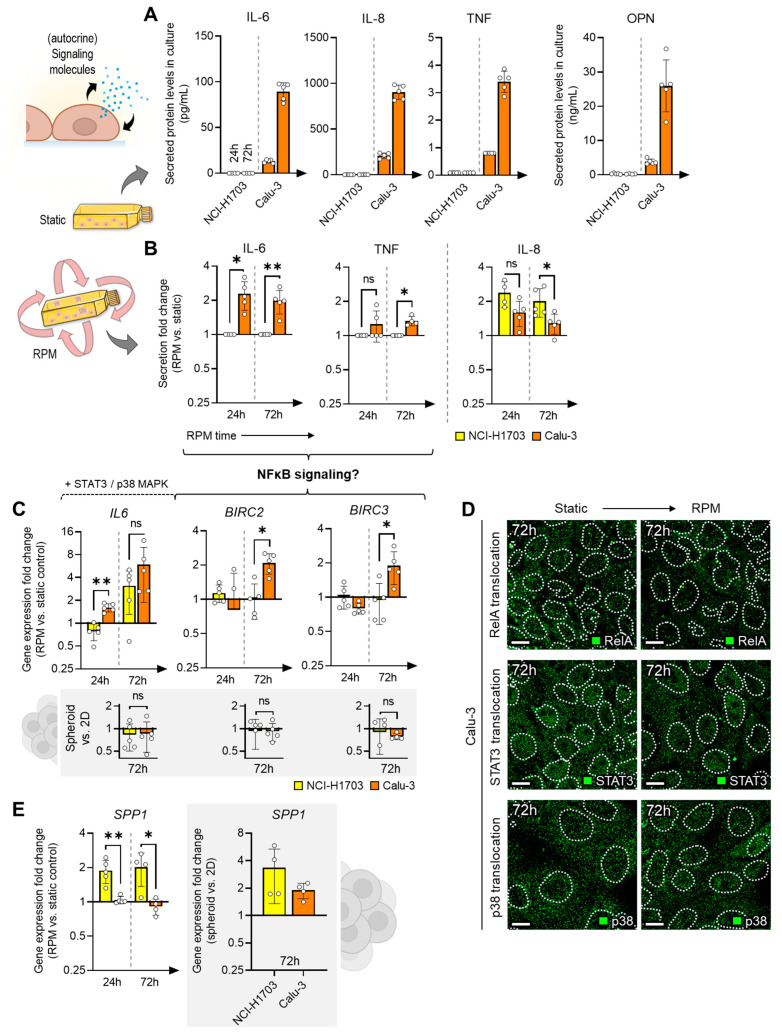
(**A**) Protein levels in the supernatant of static cell cultures of NCI-H1703 and Calu-3 after 24 and 72 h. (**B**) Effects of RPM cell culture on the content of secreted proteins. (**C**) Expression changes in NFκB target genes during RPM cell culture (*n* = 5). The small diagrams in the gray box describe the changes in gene expression in tumor spheroid cells. (**D**) Immunofluorescence of RelA, STAT3 and p38 in a 3-day culture (*n* = 4–5 for each condition; one representative picture is shown). Outlines of the nuclei as indicated using DAPI staining depicted as dashed lines. Scale bar: 10 µm. (**E**) Expression changes in *SPP1* during RPM cell culture (*n* = 4–5). Non-parametric Mann–Whitney U test * *p* ≤ 0.05, ** *p* ≤ 0.01, ^ns^ non-significant. Parts of the figure were drawn using pictures from Biorender.com and Servier Medical Art.

Possible stimuli that can affect the spheroid cells include mechanical (RPM, shear forces) and chemical (signaling molecules) influences. For the latter, a cell culture supernatant analysis was performed with a focus on (inflammatory) factors that have been associated with spheroid formation in previous RPM studies [13]. The cell culture supernatant of the Calu-3 culture generally contained much higher levels of the secreted proteins (including IL-6, IL-8, TNF, OPN) compared to the NCI-H1703 culture, which also increased over the culture period (Figure 4A). The supernatant of RPM-exposed Calu-3 cells showed even higher levels of the inflammatory markers IL-6 and TNF, while IL-8, which was elevated in most cell cultures with 3D growth, was more upregulated in the NCI-H1703 samples than in Calu-3 samples (Figure 4B). Secreted osteopontin (OPN) levels were not altered by the RPM. The increased secretion of IL-6 and TNF indicated involvement of NFκB signaling in Calu-3 cells exposed to the RPM. Indeed, we found early upregulation of the NFκB target gene *IL6* (24 h) and delayed upregulation of *BIRC2* and *BIRC3* (72 h) in Calu-3 cells from the RPM experiment (Figure 4C). This upregulation was induced by the RPM in the adherent cell population and did not change during spheroid formation (Figure 4C, gray box). However, no translocation of RelA into the nucleus of Calu-3 cells could be observed after 72 h on the RPM (Figure 4D), which rules out activation of the NFκB signaling pathway in Calu-3 cells on the RPM as a trigger for the secreted factors. Other transcription factors involved in mechanosignaling, such as STAT3 and p38, were also not increasingly translocated to the cell nucleus (Figure 4D). Therefore, the causative (mechano)signaling pathway for the observed gene regulation changes in Calu-3 cells remains unknown for now. Another observed transcriptional effect was the upregulation of *SPP1* (coding for osteopontin) in NCI-H1703 cells on the RPM (Figure 4E).

### 3.4. Calu-3 Cells Upregulate ESR1 Expression during Random Positioning

**Figure 5:**
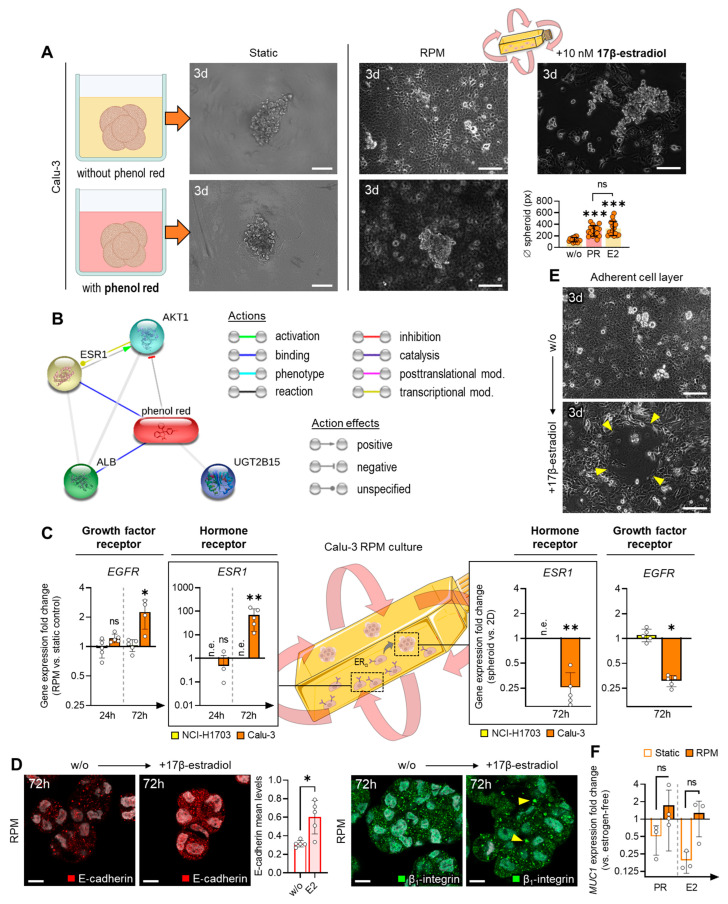
(**A**) Effect of phenol red on the spheroid formation of Calu-3 cells under static and RPM conditions after 72 h. The bar chart shows the spheroid size on the microscopic images in phenol-red-free medium (w/o), medium with phenol red (PR) and medium with 10 nM 17β-estradiol (E2). Scale bar: 200 µm. (**B**) STITCH v5.0 interaction network of phenol red in human cells. (**C**) Expression of *EGFR* and *ESR1* in two-dimensional-growing Calu-3 cells (left) and in Calu-3 spheroids (right) on the RPM (*n* = 5). n.e., not expressed. (**D**) Immunofluorescence of E-cadherin and β_1_-integrin in a 3-day culture (*n* = 5 for each condition; one representative picture is shown). Nuclei are stained with DAPI (gray). The increased formation of integrin clusters is indicated by yellow arrows. The averaged mean values of E-cadherin fluorescence per cell for each microscopic image are shown in the bar graph. Scale bar: 10 µm. (**E**) Adherent cell layer of Calu-3 cells after 3 days on an RPM without and with 10 nM 17β-estradiol. Scale bar: 200 µm. (**F**) Expression of *MUC1* in Calu-3 cells on the RPM in the presence of phenol red (PR) or 10 nM 17β-estradiol (E2) (*n* = 3–4). AKT1, Rho family-alpha serine/threonine-protein kinase; ALB, albumin; ESR1, estrogen receptor 1; ERα, estrogen receptor alpha; UGT2B15, UDP glucuronosyltransferase 2 family polypeptide B15. Non-parametric Mann–Whitney U test * *p* ≤ 0.05, ** *p* ≤ 0.01, *** *p* ≤ 0.001, ^ns^ non-significant. Parts of the figure were drawn using pictures from Biorender.com and Servier Medical Art.

Since larger Calu-3 spheroids have already been described in previous RPM studies after a three-day-rotation culture [12], we next focused on the differences to the current experiments. The main difference in our experiments was the use of a phenol-red-free medium. A comparative experiment with phenol-red-containing medium of the same composition led to the formation of NCI-H1703 spheroids and of larger Calu-3 spheroids on the RPM (Figure 5A). Since phenol red has an estrogen-like structure and can therefore bind to the estrogen receptor ERα (Figure 5B) [24], we examined the expression of *ESR1* in the RPM-exposed cells. The expression of *ESR1* was undetectable in NCI-H1703 cells, and *ESR1* was only weakly expressed in Calu-3 cells. However, we found a very strong upregulation of *ESR1* in adherent Calu-3 cells after 3 days on the RPM (Figure 5C, boxes). Although *ESR1* was lower expressed in the cells of the Calu-3 spheroids, this expression was still higher than in normal cell cultures. A similar regulatory behavior, albeit much weaker, was observed for the growth factor receptor gene *EGFR* (Figure 5C).

An immunofluorescence analysis of 3-day-old Calu-3 spheroids from the RPM showed a higher amount of E-cadherin in the presence of 10 nM 17β-estradiol (Figure 5D), which should contribute to an increased stability of the spheroids. β_1_-integrin was more clustered in spheroids exposed to estradiol (Figure 5D, yellow arrows).

To test whether phenol red is indeed the cause of the more stable Calu-3 spheroids that were formed on the RPM, 10 nM 17β-estradiol was added to phenol-red-free medium in a further random positioning experiment. In the presence of estradiol, many larger spheroids also formed after 72 h in the RPM culture (Figure 5A, right column). In these samples, the effect of spheroid formation was even more pronounced than with phenol red. The adherent cell layer was thinner in the presence of estradiol and showed intermittent gaps (Figure 5E, arrows). A possible explanation would be the trend toward an increased expression of *MUC1* in the presence of estrogens (phenol red (*p* = 0.057) and 17β-estradiol (*p* = 0.100)) in the RPM-exposed cells compared to a static culture (Figure 5F).

## 4. Discussion

Cultivation of lung carcinoma cells on the RPM also leads to tumor spheroid formation. By using different types of lung cancer in this study, it was possible to gain new and deeper insights into the complex processes of “in vitro metastasis” using the RPM.

### 4.1. Spheroid Formation

**Figure 6:**
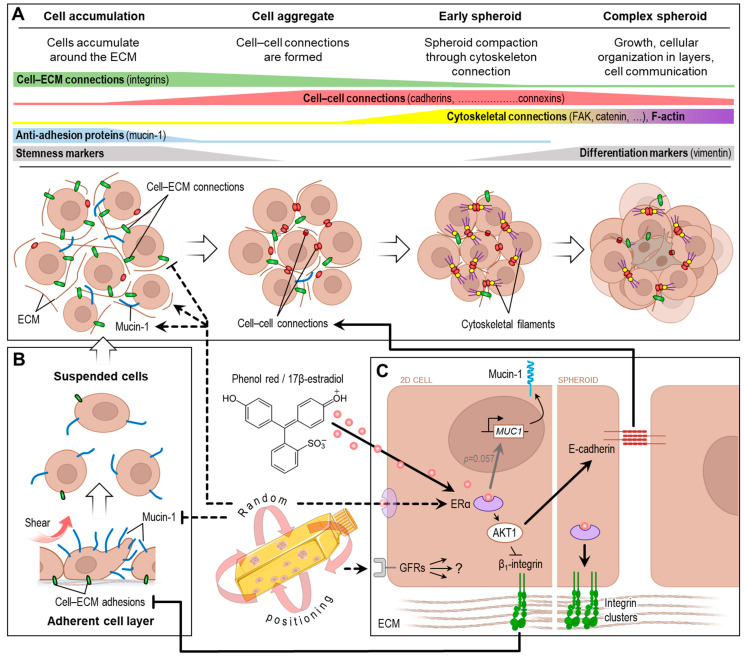
The current model of spheroid formation on a random positioning machine. (**A**) The aggregation of a loose accumulation of suspension cells into a compact, stable spheroid takes place in various stages, each of which focuses on different structural proteins. (**B**) The easy detachment of the cells from the adherent cell layer is dependent on the expression of anti-adhesive mucin-1 (blue). (**C**) Presumed molecular biological effects of ERα signaling on the formation of spheroids. Arrows indicate activation/increase, T-arrows inhibition/decrease. Dashed lines indicate the relationships that lead to few unstable Calu-3 spheroids in the RPM cell culture. Solid lines indicate the processes that lead to more and more stable Calu-3 spheroids in the presence of phenol red/estrogen on the RPM. ECM, extracellular matrix; ER, estrogen receptor; GFR, growth factor receptor. Parts of the figure were drawn using pictures from Biorender.com and Servier Medical Art.

The development of stable spheroids requires the temporally coordinated interaction of ECM components, cell–ECM and cell–cell connections (Figure 6A). Only if these conditions are met can spheroids be observed as a subpopulation of the cell culture at the end of an RPM experiment.

We observed a better spheroid-forming ability of NCI-H1703 squamous cell carcinoma cells compared to Calu-3 adenocarcinoma cells during normal RPM operation. This is an interesting result, as Ahn et al. [18] had previously observed a stronger proliferation and migration of A549 adenocarcinoma cells compared to NCI-H1703 cells on the RPM. It is quite possible that cancer cell migration in the RPM model does not correlate with spheroid formation. What we could confirm in our experiments was a different expression of adhesion and anti-adhesion proteins in both cell types, the “glue mixture” that holds them together. Compared to NCI-H1703 cells, Calu-3 cells exhibited higher levels of adhesion proteins and lower amounts of the anti-adhesion protein mucin-1, which also plays an important role in cancer metastasis [25,26]. The key role of mucin-1 in RPM-induced spheroid formation (Figure 6B) suggested in the work of Melnik et al. [14] was also confirmed in this study. Calu-3 cells in two-dimensional culture showed an even lower mucin-1 expression on the RPM, which may be related to their lower tendency to form spheroids during rotation (more pronounced 2D cell layer). Why Calu-3 cells downregulate mucin-1, while other cell types (such as Nthy-ori 3-1 thyrocytes) upregulate it during random positioning [14], cannot be answered at present.

During our studies, it became apparent that the stability of the Calu-3 spheroids formed was so low that they disintegrated due to the shear forces of the RPM. Consistent with this, we found an upregulation of *MUC1* and a strong downregulation of *ITGB1* expression in Calu-3 spheroid cells on the RPM. β_1_-integrin is one of the most important factors mediating cohesion in early cell aggregates [27,28,29,30]. The cell aggregates are then stabilized by cell–cell junctions (cadherins, connexins and possibly also tight junction proteins). One stage later, the cytoskeleton is coupled to the cell–cell junctions via connecting proteins (focal adhesions, etc.) (Figure 6A). At the protein level, it was confirmed that Calu-3 tumor spheroids have fewer adhesion proteins (β_1_-integrin, E-cadherin) and instead express much more mucin-1 and laminin. Since mucin-1 was found mainly around smaller subaggregates (Figure 3D), it is possible that these cell clusters aggregate through laminin but do not coalesce into larger stable spheroids due to the anti-adhesion effect of mucin-1 (in combination with lower-expressed β_1_-integrin and E-cadherin). This could explain the cauliflower-like structure of Calu-3 spheroids.

In our 3-day experiments, we were only able to observe spheroid development up to the early aggregation phase, as we could not yet observe cytoskeletal linkage or mature focal adhesions. Looking at the most important proteins present in 3-day-old cell aggregates, the Calu-3 spheroids were probably still in the (first) aggregation phase, while the NCI-H1703 spheroids were already one developmental step further (see Figure 6A). Whether the spheroid formation (detachment) of the Calu-3 cells started later due to less mucin-1 or whether the aggregation process in Calu-3 cells generally takes longer remains to be investigated. An upregulation of *CDH1* over a period of 3 days was also described by Baghoum et al. [31] for clinorotated A549 cells. In addition, the levels of *TJP1* (tight junction protein 1, ZO-1) mRNA were increased after 3 days of rotation. Unfortunately, it is not clear from this study whether only the spheroids were analyzed (which would then have been in the aggregation phase) or whether it was a whole culture analysis.

We also found increased proinflammatory signaling molecules in the cell culture supernatant of Calu-3 cells exposed to the RPM. However, an involvement of the NFκB signaling pathway in the preparation of spheroid formation on the RPM, as described in breast cancer cells [32], can be excluded for the two lung carcinoma cell lines investigated.

NCI-H1703 cells also showed transcriptional upregulation of *SPP1* on the RPM. In breast cancer, osteopontin is known to promote 3D growth [33]. Therefore, *SPP1* expression is also often investigated in RPM studies (recently summarized in [34]). However, the extent to which it interferes with RPM-induced tumor spheroid formation is unclear, especially since no elevated levels of osteopontin were found in the NCI-H1703 randomly positioned cell cultures.

In RPM-cultured cancer cells, several factors are altered at the transcriptome, proteome and secretome level. Not all of them contribute to the phenomenon of spheroid formation. The assignment of RPM effects at the molecular level to the different biological and phenotypic processes is one of the challenges of the coming years.

### 4.2. Random Positioning and Estrogen Receptor

An interesting, albeit unexpected, result of this work was the influence of the *ESR1* expression of Calu-3 cells via the RPM. It is already known from breast cancer research that the transcriptional regulation of *ESR1* can also be controlled by various extrinsic factors [35]. Hormones are important signaling molecules in the body, and a change in hormone sensitivity has a major impact on the biology of cells. In cancer cells in particular, hormone dependency often determines the progression and treatability of the disease [36]. Laboratory results, for example, have indicated a carcinogenic effect of estrogens in female lung cancer [37]. Conversely, this finding also means that the RPM (“simulated microgravity”; s-µg) cell culture can influence the proliferation and drug sensitivity of cancer cells by increasing ER expression. This should be considered when ER-positive cells are used together with estrogen-like additives (e.g., phenol red in human cell culture medium) in RPM studies. Observations such as altered proliferation [38,39,40,41,42,43] and chemosensitivity [44,45,46,47,48] have often been described for rotating (s-µg) experiments with cancer cells and may be related to hormone receptor signaling pathways, among others. As a possible consequence of *ESR1* upregulation in this study, we were able to show that estrogens and phenol red have an influence on the spheroid stability (3D growth) of ERα-positive Calu-3 cells on the RPM. It has already been described for breast cancer cells that estrogens contribute to the increased formation of desmosomes via ERα and thus to cell–cell adhesion, making them less invasive [49]. An important step in this process is the AKT1-mediated downregulation of β_1_-integrin and the upregulation of E-cadherin (Figure 6C) [50]. In the presence of 17β-estradiol, we indeed found more E-cadherin in the Calu-3 spheroids, as well as an increased clustering of β_1_-integrin, which also indicates a more stable adhesion [51]. In addition, *MUC1* expression was increased in the presence of estrogens, as has already been described for breast cancer cells [52,53]. Upregulation of *ESR1* and *PGR1* was previously described only once by Kopp et al. [11] for MCF-7 cells in a 5-day RPM cell culture. In this study, in which a phenol-red-containing medium was also used, the formation of duct-like spheroids was observed. Increased ERα expression was also reported in MCF-7 cells exposed to a continuously spinning rotary cell culture system [54]. The authors found evidence that ERα may play an important role in protecting cells from oxidative stress damage under s-µg. However, it should be noted that the cells were encapsulated in a matrix and the approach is therefore not completely comparable to our experiments.

Due to the limited data available, it is not yet possible to assess the extent to which an RPM can simulate the effects of real (space) microgravity on the estrogen sensitivity of human cells. Estrogens are often described to change in vivo during space travel or microgravity analogy studies [55]. Some studies report a decrease of estrogen levels [56], which in turn could be compensated by an increased estrogen sensitivity of body cells. On the other hand, there is currently little research on how estrogen signaling and related pathways change during spaceflight. In humans, one study reported enriched estrogen signaling pathways and an upregulation of *ESR1* within 2 months postflight followed by a downregulation in the 3rd month [57]. The data on estrogen signaling in rodents are conflicting. One study reports a downregulation of *ESR1* and *ESR2* in the uterus of mice [58], with another study finding no alterations in estrogen receptor expression in the ovaries of mice after being flown to space [59]. Further studies are needed in this area.

### 4.3. Random Positioning and Growth Factor Receptors

We further observed an increase in *EGFR* transcription in two-dimensional-growing Calu-3 cells after 3 days on the RPM. The influence of random positioning on the transcription of growth factor receptor genes in cancer cells has already been mentioned in earlier RPM studies, but the literature of recent years does not provide a consistent picture here. Hybel et al. [3] found an increased expression of *KDR*/*FLK1* in RPM-exposed PC-3 prostate cells after 3 days and of *FLT1* after 5 days, which would be consistent with the increase in *EGFR* expression we observed. However, a decrease in *EGFR* expression was observed in MCF-7 breast cancer cells after 14 days on the RPM [60]. Melnik et al. [61] also found a reduction in *EGFR* expression after 3 days of rotation in FTC-133 thyroid cancer cells. Thus, the transcriptional control of growth factor receptor expression during random positioning appears to be cell-type dependent or subject to a more complex regulatory pattern, which requires further investigation. It is also unclear whether these growth factors and their signaling pathways contribute to the process of spheroid formation (Figure 6C, question mark), as tumor spheroids were clearly formed in all the studies mentioned above.

## 5. Conclusions

The comparison of the RPM-induced tumor spheroid formation of Calu-3 and NCI-H1703 lung carcinomas revealed the complex processes during random positioning. In agreement with previous work, mucin-1 again influenced the detachment of the adherent growing cancer cells by the flow shear occurring on the RPM and thus the quantitative ratio of the formed subpopulations (cell layer vs. spheroids) at the end of the experiment. Calu-3 data indicated that mucin-1 is not only an important factor in the initiation of spheroid formation (detachment) but also for the stability of the subsequently formed spheroids (mainly mediated via cell–cell junctions), a prerequisite for their integrity under the shear stress conditions on the RPM. We have now been able to show that random positioning can increase the estrogen sensitivity of a cell culture and that estrogens (as well as phenol red) affect the expression of E-cadherin and mucin-1, which in turn influences spheroid stability. Accordingly, the spheroid formation induced by the RPM depends not only on cell-type-specific properties and the mechanical influences of random positioning on the cells but also partly on the chemical composition of the medium used during the experiments.

## Data Availability

The data presented in this study are available in the electronic Supplementary Materials. The complete raw data are available on request.

## Supplementary Materials

The following supporting information can be downloaded at https://www.mdpi.com/article/10.3390/biom14101292/s1, Table S1: Primer sequences for quantitative real-time PCR; Table S2: Antibodies used for immunofluorescence analyses.
