# Supplementary material for: The Formation of Stable Lung Tumor Spheroids during Random Positioning Involves Increased Estrogen Sensitivity
Source: Biomolecules. 2024 Oct 12;14(10):1292. doi: 10.3390/biom14101292 (PMC11506229; doi:10.3390/biom14101292)
Supplement: Supplementary file 1 [file biomolecules-14-01292-s001.zip › biomolecules-3221273-supplementary.pdf]

Table S1. Primer sequences for quantitative real-time PCR.

Gene	Primer Name	Sequence (5′→3′)
18S rRNA	18-F	GGAGCCTGCGGCTTAATTT
	18-R	CAACTAAGAACGGCCATGCA
BIRC2	BIRC2-F	GCTTTTGTTGTGATGGTGGCT
	BIRC2-R	ACTCACACCTTGGAAACCACT
BIRC3	BIRC3-F	TGCTGTGATGGTGGACTCAG
	BIRC3-R	ACTCACACCTTGGAAACCACT
CDH1	CDH1-F	GCTGGACCGAGAGAGTTTCC
	CDH1-R	CAGCTGTTGCTGTTGTGCTT
EGFR	EGFR-F	TTGCCGCAAAGTGTGTAACG
	EGFR-R	GAGATCGCCACTGATGGAGG
ESR1	ESR1-F	TTCAAGAGAAGTATTCAAGGACATAACG
	ESR1-R	TCGTATCCCACCTTTCATCATTC
GJA1	GJA1-F	GACAGGTCTGAGTGCCTGAAC
	GJA1-R	TTGCCTGGGCACCACTCTTT
ICAM1	ICAM1-F	CGGCTGACGTGTGCAGTAAT
	ICAM1-R	CTTCTGAGACCTCTGGCTTCGT
IL6	IL6-F	CGGGAACGAAAGAGAAGCTCTA
	IL6-R	GAGCAGCCCCAGGGAGAA
ITGA4	ITGA4-F	CCAGCTGGGTAGCCCTAATG
	ITGA4-R	CCTGGCTGTCTGGAAAGTGT
ITGB1	ITGB1-F	GAAAACAGCGCATATCTGGAAATT
	ITGB1-R	CAGCCAATCAGTGATCCACAA
MUC1	MUC1-F	CCTCACAGTGCTTACAGTTGTT
	MUC1-R	GCTGGGCACTGAACTTCTCT
SPP1	OPN-F	CGAGGTGATAGTGTGGTTTATGGA
	OPN-R	CGTCTGTAGCATCAGGGTACTG

Table S2. Antibodies used for immunofluorescence analyses.

**Antibody** (anti-…)	**Company, Product Nr.**	**Species**	**Dilution IF**
MUC1	Invitrogen, #MA1-35039	Mouse	1:100
FAK	Abcam, #ab40794	Rabbit	1:200
STAT3	Cell Signaling, #9139	Mouse	1:200
p38 MAPK	Invitrogen, #MA5-15116	Mouse	1:1000
NFκB p65 (RelA)	Invitrogen, #51-0500)	Rabbit	1:500
E-cadherin	Invitrogen, #MA5-11496	Mouse	1:100
Integrin-β1	Novus Biologicals, #NB110-57123	Rabbit	1:100
Laminin	Invitrogen, #PA1-16730	Rabbit	1:100

**Secondary antibody**	**Labelling**	**Company, Product Nr.**	**Dilution**
anti-Mouse	IF488	Abcam, #ab150113	1:200
anti- Rabbit	IF488	Abcam, #ab150077	1:200

AF, Alexa Fluor; IF, immunofluorescence.
